# Effect of cooking modes on quality and flavor characteristic in *Clitocybe squamulose* chicken soup

**DOI:** 10.3389/fnut.2022.1048352

**Published:** 2022-11-15

**Authors:** Jing Lai, Ruiyun Wu, Ji Wang, Ying Wang, Xin Zhang, Liyuan Zhou, Yingchun Zhu

**Affiliations:** ^1^College of Food Science and Engineering, Shanxi Agricultural University, Jinzhong, China; ^2^College of Food Science and Biotechnology, Tianjin Agricultural University, Tianjin, China

**Keywords:** chicken soup, cooking mode, quality, sensory evaluation, flavor characteristics, orthogonal partial least squares discriminant analysis (OPLS-DA)

## Abstract

The effects of cooking modes [cooking in stainless-steel pot (SS), ceramic pot (CP), and electrical ceramic stewpot (EC) with different stewing time] on chemical compositions, whiteness, 5′-nucleotides, fatty acids (FAs), sensory quality and flavor substances in chicken soup added *Clitocybe squamulose (Pers.) Kumm* (a natural edible fungus) were investigated. The results showed that CP chicken soup had higher soluble solid matter (5.83 g/100 mL), total sugar (2.38 mg/mL), crude protein (7.58 g/100 g), and 5′-nucleotides (325.53 mg/mL) than EC and SS chicken soups. 48 volatile flavor compounds, mainly aldehydes and alkanes, were found by gas chromatography-mass spectrometry (GC-MS), and the characteristic flavor substances were identified by Principal component analysis (PCA) and orthogonal partial least squares discrimination analysis (OPLS-DA). Hexanal, (E,E)-2,4-decadienal and 3-methyl-hexadecane were the most abundant differential volatile compounds in the CP chicken soup. Additionally, the results of sensory evaluation showed that the chicken soup cooked in CP had the higher values of aroma, taste, and overall acceptability. Our results indicate that CP mode might be the best option for cooking chicken soup. This study provides a new perspective in the improvement of the quality and flavor of chicken soup by using an appropriate cooking mode. Theoretical support for the use of various cooking modes is also discussed to improve the quality of chicken soup at home and in the industry.

## Introduction

Chicken soup is highly appreciated because of its unique flavor (1). Moreover, the formation of flavor substances in chicken soup is a complex process. During the stewing procedures, non-volatile compound precursors such as free amino acids, reducing sugars, peptides, nucleotides, and unsaturated fatty acids (Fas) can be fully dissolved in the soup, thus conferring the soup sweetness, saltiness, and umami (2). Meanwhile, volatile compound precursor such as aldehydes, alcohols, and ketones are also released through the Maillard reaction and fatty acid oxidation, which provides the special soup flavor (3). With the improvement of people’s living standards, pursuit of chicken soup cease to be limited to taste but has become more focused on the development of an especial nutritious concoction. The addition of mushrooms to the chicken soup, which combines the unique aroma and rich nutrients of mushrooms, greatly enhances the nutritional value of this food (4). *Clitocybe squamulose (Pers.) Kumm* is a natural edible mushroom, which is widely distributed in Wutai Mountain Ecological Park, Shanxi Province, China. Its fruiting bodies have a firm and tender texture, unique flavor, and rich nutrition (5). Simmering *Clitocybe squamulose* in chicken soup is more effective in promoting its quality and the formation of volatile flavor substances.

The existing studies mainly focus on the effect of temperature, time, and salt addition methods on the formation of flavor substances during chicken soup cooking (6–8). It should be noted that different soup cooking methods may change the flavor substances and nutrients of the soup. A previous study (9) has reported that four cooking methods (autoclaving, microwaving, sous vide, and stewing) had characteristic effects on the properties of mushroom (*Hypsizygus marmoreus*) soup and that cooking improved the nutritional value of mushrooms by increasing the release macro-nutrients and micro-nutrients. Sous vide increased the nucleotide content, which was decreased by other methods. This method was also the best technique for increasing polyphenol and flavor compounds. Zou et al. (10) have suggested that pork rib soup and silkie chicken soup have better flavor and nutritional value by four-stage stewing than by boiling and steaming, respectively.

There are various cooking options available for chicken soup. The traditional way of cooking chicken soup is to use a ceramic pot (CP) for cooking, which can confer the special flavor to chicken soup and popularity. However, an ordinary stainless-steel pot (SS) is a common choice in daily life for simmering chicken soup (7, 11, 12). A pressure cooker can greatly reduce the simmering time of chicken soup, but the resulting taste and flavor are poor (13). Nowadays, with the development of the food industry, electrical ceramic stewpot (EC) is gradually occupying the market (14, 15). However, little attention has been devoted to the effect of the cooking mode (different pots and stewing modes) to preparing chicken soup on chicken soup flavor, and the mechanisms by which cooking methods affect the nutritional quality and flavor of chicken soup are unclear.

This study aimed to analyze the soluble solid matter, total sugars, crude protein, total lipid, whiteness, 5′-nucleotides, sensory quality of *Clitocybe squamulose* chicken soups and characterize flavor compounds by gas chromatography-mass spectrometry (GC-MS). Moreover, volatile flavor substance composition in chicken soup cooked with SS, CP and EC was compared by principal component analysis (PCA) and orthogonal partial least squares discriminant analysis (OPLS-DA). Our results will provide an insight into the key characteristic flavor substances in chicken soup and lay foundation for the studies of quality chicken soup cooked with different cookwares.

## Materials and methods

### Materials

SS (6.8 L, SZ26B5) and EC (6 L, thickness = 0.56 cm, DG60YC806) were purchased from Supor Household Electric Appliance Co., Ltd. (Zhejiang, China). CP (8 L, thickness = 0.81 cm) was purchased from Shanxi Pingding Casserole Co., Ltd. (Shanxi, China). Closed-type electric furnace (FL-2Y) was purchased from Lichen Science and Technology Co., Ltd. (Shanghai, China).

*Clitocybe squamulose (Pers.) Kumm* was supplied by the Shanxi Edible Mushroom Engineering Technology Research Centre and dried in an oven at 35°C (The contents of crude lipid, crude fiber, carbohydrate, crude protein, ash, and moisture in *Clitocybe squamulose* is 3.27, 8.16, 40.96, 38.57, 3.36, and 5.68%, respectively). The internal standard 1,2-dichlorobenzene was of Chromatographic purity, and it was from Sigma-Aldrich Co., Ltd. (Shanghai, China). To measure the retention indices (RI), n-alkanes (C7-C40) was purchased from Sinopharm Chemical Reagent Co., Ltd. (Shanghai, China). The other reagents and chemicals were provided by Sinopharm Chemical Reagent Co., Ltd. (Shanghai, China).

### Cooking of *Clitocybe squamulose* chicken soup and sample preparation

Chicken soup production was optimized according to the procedures we have previously reported (16). The three-yellow chickens were provided by College of Veterinary, Shanxi Agricultural University (Taigu, Jinzhong, China), fed with a commercial diet for 5 months, then slaughtered with neck, feet, and visible muscular fat removed. The mean carcass weights were between 900 and 1,000 g. The chicken were washed and air dried before weighing. Thirty chickens were chopped into a uniform of about 3 ± 0.2 cm and mixed thoroughly. The chicken soup was prepared with the addition of *Clitocybe squamulose*, salt, and ginger at the carcass/ *Clitocybe squamulose*/salt/ginger/water weight ratio of 100:6:1.5:3:300. In this study, three chicken soup (SS group, CP group, and EC group) were prepared by using different pots and stewing modes. SS group referred to soup prepared in SS, CP group represented soup cooked in CP, and EC group indicated the soup prepared in EC. SS and CP were heated on the closed-type electric furnace; the third group was prepared in EC by choosing its Soup Mode, and the processing parameters were as follows. Firstly, water, meat and other ingredients were heated from 18.0 to 96.5°C for 15 min (SS), 60 min (CP) and 175 min (EC), then stewed at 96.5 ± 1.0°C, the total cooking time is 330 min for every type of chicken soup. The temperature was measured with data acquisition instrument (Agilent 34970A, Agilent Technologies Co., Ltd., Santa Rosa, CA, USA), and the specific sample temperature profile is shown in Supplementary Figure 1. The water level before heating was marked as the initial water level. During the stewing process, the water level was checked every 30 min, and the boiling water was added to maintain the initial water level. The amount of extra added water is 1,189 g (SS), 127 g (CP) and 586 g (EC), respectively. Finally, the chicken soup was filtered through four layers of cotton gauze to remove the solid residues, and the filtered soup was stored at −20°C for subsequent analyses.

### Determination of soluble solid matter

The content of soluble solid matter was measured, as previously reported (17) with slight modification. Briefly, samples were filtered using a filter paper (No. 101, φ15 cm, Beimu Pulp Paper Co., Ltd., Hangzhou, Zhejiang, China). The 10 mL filtrate was added with distilled water to reach 100 mL volume in volumetric flask. The crucible was dried in an oven (DHG9140A, Shanghai Huitai Instrument Manufacturing Co., Ltd., Shanghai, China) at 103°C until the crucible weight difference between the two weightings was less than 2 mg to obtain constant weight. Then, 5 mL sample dilutions was added in a crucible and dried in an oven at 103°C for 4 h until constant mass was obtained. The content of soluble solid matter was calculated according to the following formula:


$$X=\frac{m_{2}-m_{1}}{\left(\frac{10}{100}\right)\times 5}\times 100$$

Where X represented the content of soluble solid matter in sample (g/100 mL); m_1_ represented the mass of the crucible (g); m_2_ represented the total mass of the soluble solids and crucible after 4 h of drying (g).

### Determination of total sugar

The content of total sugar was determined according to phenol-sulfuric acid method (18). Anhydrous glucose was used as the standard. Linear regression equation (y = 6.1602x + 0.0042, *R*^2^ = 0.9971) was used to calculate the content of total sugar content, expressed as mg/mL.

### Determination of crude protein

The crude protein content in chicken soup samples was determined through Kjeldahl digestion (N × 6.25) (19). The 15 g chicken soup was added to a nitrogen tube, and then 0.4 g copper (II) sulfate pentahydrate, 6 g potassium sulfate, and 20 mL sulfuric acid were added. The digestion was then conducted on graphite digestion instrument (Hanon SH220, Hanon Instrument Co., Ltd. Jinan, Shandong) as follows: 120°C for 30 min, 240°C for 30 min, 360°C for 1 h, 420°C for 1 h, followed by cooling to room temperature. Nitrogen determination was performed with an automatic Kjeldahl nitrogen analyzer (ZDDN-II, Tuopu Instrument Co., Ltd., Hangzhou, Zhejiang).

### Detection of total lipid

The total lipid content was determined, as previously described (20). Briefly, 7 mL chicken soup was added into 90 mL chloroform-methanol (2:1, v/v) solution and oscillated at 45°C for 2 h. The 30 mL NaCl solution (0.9%, w/w) was added and mixed evenly. The chloroform was removed using a rotary evaporator (RE-52AA, Shanghai Yarong Biochemistry Instrument Factory, Shanghai, China) at 40°C. The remaining material was weighed with an electronic analytical balance (CP114, Ohaus International Trading Co., Ltd., Changzhou, Jiangsu, China) and was taken as the total lipid, which was expressed as %.

### Detection of fatty acids

Total lipids of chicken soup were extracted by homogenization with chloroform/methanol (2:1, v/v), as described by Xun et al. (21). The extracts were then mixed with 10 mL sodium chloride solution (0.9%, w/w) and shaken vigorously for 5 min. After that, the mixture was left to stand at 4that, t h, and the lower layer was collected and taken to a new centrifuge tube and blown dry with nitrogen. Mixtures of lipid and 2 mL sodium hydroxide methanol (0.5 mol/L) were kept in a water bath at 65in metha min for transesterification, then 2 mL boron trifluoride methanol (15%, w/w) were added and further heated at 65°C for 30 min. Following cooling, n-hexane (2 mL) and saturated sodium chloride solution (2 mL) was added. At last, the mixture was shaken, extracted, stood still to stratify. The upper layer was harvested for GC (GC-2030, Thermo Fisher Inc., Waltham, USA) analysis. GC was equipped with a capillary column (Agilent SP-2560, 100 m × 0.25 μm × 0.2 μm) and a flame ionization detector (FID, Thermo Fisher Inc., Waltham, USA). The mixed standard solutions of 37 fatty acid methyl esters were prepared into mixed standard solutions. FAs identification was made by comparing the relative retention times of fatty acid ethyl ester (FAMEs) peaks from samples with standards.

### Measurement of whiteness

The color measurement for the chicken soup was performed according to the CIE L*, a* and b* color system (CM-5 Colourimeter, Konica Minolta Sensing Inc., Osaka, Japan) with a measurement area diameter of 11 mm, and soup was illuminated by D65 standard illuminant (pulsed xenon lamp). The whiteness of chicken soup was calculated using the following formula:


$$W=100-{\left[{\left(100-L^{*}\right)}^{2}+a^{*2}+b^{*2}\right]}^{\frac{1}{2}}$$

Where L* indicated brightness; a* stood for red-greenness, and b* represented yellow-blueness.

### Detection of 5′-nucleotides

The nucleotide content in samples was measured according to the method described by Zhang et al. (15) with slight modification. Chicken soup (10 g) was centrifuged at 5,000 g for 15 min at 4entrifuged at 5,n. Chicken soupa 0.22-μm membrane before analyses. Ten microliters of the filtrate were injected into an HPLC system (Agilent 1260ALS, USA) with the detection wavelength set at 254 nm. The separation of nucleotides was achieved by a Venusil ASB C18 column (4.6 mm × 250 mm × 5 μm) and its temperature was set at 30°C. The mobile phase contained methanol, distilled water and phosphoric acid (ratio 16:400:1, v:v:v) with a flow rate of 1 mL/min. The 5′-nucleotides were quantified based on the external calibration curves, expressed as mg/L.

### Sensory evaluation

The sensory evaluation was performed in the sensory analysis laboratory (College of Food Science and Engineering, Shanxi Agricultural University, Jinzhong, Shanxi, China) by ten experienced postgraduate students majoring in food science (4 males and 6 females with an average age of 24.5 years). The 60 mL of the chicken soup sample was placed into a transparent plastic cup which was coded with a 3-digit random number, and the soup temperature was maintained at 45°C to avoid the influence of temperature difference on the flavor attributes. The sensory evaluation was conducted based on a 15-point scale, and the assessors scored for color (15 = pale yellow; 1 = dark and dull), taste (15 = extremely desirable; 1 = extremely undesirable), aroma (15 = extremely desirable; 1 = extremely undesirable), clarity (15 = clear; 1 = cloudy), oily (15 = non-greasy; 1 = greasy), and overall acceptability (15 = high; 1 = low).

### Determination of volatile compounds

The volatile compounds were analyzed by headspace SPME (solid-phase micro-extraction) and GC-MS (Trace ISQ, Thermo Fisher Scientific Co., Ltd., Shanghai, China). The detailed analysis processes were as follows. Each soup sample (7 mL) was placed into a 20 mL headspace sample vial containing 1.0 g of sodium chloride, and then 5 μL 1,2-dichlorobenzene (100 μg in 1 mL of hexane) was added to each headspace vial as internal standard. The volatile compounds were detected according to the previously reported method with some modifications (22). Volatile compounds were identified by matching their mass spectra with the NIST mass spectrum library (National Institute of Standards and Technology, Gaithersburg, MD, USA) with similarity > 80% as standards by using the Trace Finder software (Thermo Fisher Scientific, Palo Alto, CA, USA). The relative content of each volatile compound was calculated by comparing the peak area of the compound with that of the internal standard according to the following formula:


$$C=\frac{A_{X}\times C_{0}\times V\times 1000}{A_{0}\times m}$$

Where C and C_0_ represented the concentration of volatile compounds (μg/kg) and internal standard (μg/μL), respectively; Ax and A_0_ indicated the peak area of volatile compounds and internal standard, respectively (mAu⋅s); V represented the volume of internal standard added to the sample (μL); and m represented the mass of sample (g).

### Statistical analysis

All the data were expressed as mean ± SE (standard error) of three independent replicates. Statistical analysis was performed using one-way analysis of variance (ANOVA) and Duncan’s multiple range tests in the SPSS software (version 19.0, SPSS, Inc., Chicago, IL, USA). The heat maps of volatile compounds were drawn by TBtools. PCA and OPLS-DA of the volatile compounds were performed using the software SIMACA 14.1 (Sweden). The compounds with variable importance in the projection (VIP) score > 1, *p* (corr) > 0.8 in the OPLS-DA analysis and a *p*-value < 0.05 in the ANOVA were identified as significantly differential compounds among all chicken soup samples.

## Results and discussion

### The content of soluble solid matters

**FIGURE 1:**
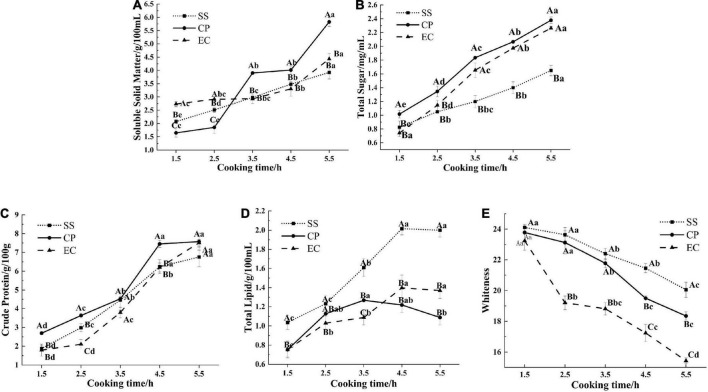
Effect of cooking mode on soluble solid matters content **(A)**, total sugars content **(B)**, crude protein content **(C)**, total lipids content **(D)** and whiteness **(E)** of *Clitocybe squamulose* chicken soup. SS, *Clitocybe squamulose* chicken soup prepared in stainless-steel pot mode; CP, *Clitocybe squamulose* chicken soup prepared in ceramic pot mode; EC, *Clitocybe squamulose* chicken soup prepared in electrical ceramic stewpot mode. The uppercase letters show differences between cooking modes per time point, and lowercase letters show differences during cooking time per cooking mode.

Soluble solid matter is one of the important indicators to assess the quality of soup, and it also reflects the overall dissolution of nutrients and flavor substances (23). During cooking, the soluble solid matters in the soup are mainly derived from amino acids, minerals, glycogen, and vitamins, and other soluble substances since these substances (19) can be released from chicken meat tissues and *Clitocybe squamulose* rapidly. The release of such chemicals can increase the soluble solid matter concentrations in the soup. In the current study, the soluble solid matter content of the soup increased with cooking time in all groups (Figure 1A). The CP group had the fastest rate of increase and a significantly higher soluble solid content than the other two groups at 3.5 h of cooking (*P* < 0.05). At the end of cooking, the soluble solid contents of the SS and EC groups were 3.92 and 4.43 g/100 mL, respectively (*P* < 0.05), whilst the CP group reached 5.83 g/100 mL, indicating a 2.54-fold increase over 4 h. This result might be due to the fact that the CP was made of ceramics composed of quartz, feldspar, clay and other raw materials. This pot had many micro-pores in its liner, leading to a rough surface with good aeration, desirable adsorption, and uniform heat transfer (14). Therefore, soluble solid materials were easily produced and released in the chicken soup. Compared with the CP group, the EC group heated up more slowly and remained boiling for a shorter duration. These phenomena limited the release of soluble solids from the ingredients into the soup, resulting in a lower soluble solid content in the EC group than in the CP group at the end of boiling. By contrast, the SS was composed of dense metal particles, which were excellent conductors of heat, and had rapid heat transfer and fast water evaporation with great loss of soluble solids (24).

### The content of total sugars

Sugar, as an indispensable nutrient in soup, not only enhances the nutritional value of the soup, but also confers the soup rich flavor characteristics (25). Within 330 min, the chicken and *Clitocybe squamulose* are boiled, and the glycogen and the other chemicals in the chicken are also released into the soup, thus causing the total sugar concentrations to increase (20). The total sugars were increasing which was consistent with the dissolution in *Salmo salar* and *Aristichthys nobilis* head soups (17). The total sugar contents of the CP (2.38 mg/mL) and EC (2.26 mg/mL) groups were significantly higher than that of the SS group (1.65 mg/mL, *P* < 0.05, Figure 1B). This result was probably due to the longer heating process that precedes CP and EC soups, which destroyed the fibrous cells of the chicken and structure of the *Clitocybe squamulose* tissues, thereby releasing sugars. Moreover, the CP and EC had micro-porous structures on their liners, which tended to generate swirls and higher pressure (26) when the soup collided with the liner. This phenomenon facilitated the migration of sugars from the ingredients into the soup.

### The content of crude proteins

The crude protein in chicken soup mainly includes chondroitin, collagen, and free amino acids (27). The cooking can hydrolyze protein and increase carbohydrates in the soup, resulting in Maillard reactions (28, 29). At the end of cooking, the crude protein content was higher for CP and EC groups at 7.58 and 7.51 g/100 g respectively, while the content of SS group was lower at 6.76 g/100 g (Figure 1C, *P* < 0.05). This result might be due to the special material of the CP with its good thermal insulation properties, which caused a higher boiling temperature and facilitated the dissolution of the crude protein (28). However, the high heat intensity of the SS tended to cause thermal denaturation of the myogenic fibrous proteins in the muscle, leading to aggregated contraction and hindering the release of protein. In addition, the heating process of the SS chicken soup was only 15 min, resulting in a large amount of water added during the longer stewing process, which also contributed to the low crude protein content.

### The content of total lipids

**FIGURE 2:**
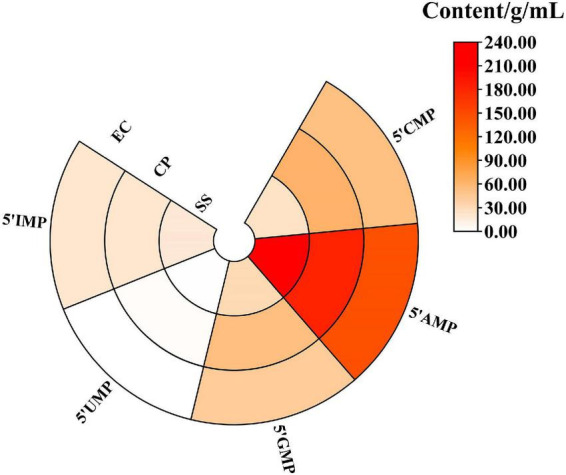
Effect of cooking mode on 5′-nucleotides of *Clitocybe squamulose* chicken soup.

During cooking process, the fat in the chicken meat is continuously released into the soup, contributing to the wonderful flavor, but high lipid content in the soup can led to greasy taste and excessively greasy flavor, which affected the sensory quality of chicken soup (20). Our results showed (Figure 1D) that cooking time had a significant effect (*P* < 0.05) on the total lipid content of the chicken soup. This content tended to increase and then decrease slightly during the cooking process. During the first stage, the fat in the subcutaneous tissue of the chicken migrated into the soup because of the high temperature, thereby increasing the lipid content in the soup. The decreased lipid content during the later stage may have been caused by the decomposition as a result of prolonged heat treatment (30). In the current study, the lipid contents in the CP and EC groups (0.75 and 1.40 g/100 mL) were significantly lower than that in the SS group (2.02 g/100 mL) after 4.5 h of cooking (*P* < 0.05, Figure 2). This phenomenon was probably due to the micro-porous structures of the liners of the CP and EC. Such structure tended to generate higher pressure when the soup collided with the liner, resulting in a micro-vortex of soup. During the swirling process, large oil droplets collided and impacted, leading to more numerous and smaller particle sizes (31) and facilitating the penetration to the cavity structure of the *Clitocybe squamulose*.

### Whiteness

Generally, color is an important quality criterion for food, especially for soup. The whiteness in the soups cooked in different cooking mode were shown in Figure 1E. In the *Clitocybe squamulose* chicken soup, the brown *Clitocybe squamulose* (32) has been boiled for a long time, thereby causing its tissues to be decomposed and its pigments to be continuously released into the soup, eventually resulting in a decrease in the whiteness value. The whiteness value was significantly different (*P* < 0.05) among chicken soups prepared with different cooking modes. As shown in Figure 1E that the whiteness value in EC group was significantly lower than that in SS group and CP group. Among the three cooking modes, the modes of EC and CP made the *Clitocybe squamulose* better decomposed and fully dissolved, ending up with a lower whiteness value.

The density and rheology of the chicken soup were examined as a response to the physical properties of the soup (Supplementary Figure 2). The density values, viscosity value and shear stress value of the CP chicken soup were the greater compared to the SS and EC groups. The results indicated that CP chicken soup has the superb physical properties.

### The content of 5′-nucleotides

The 5′-nucleotide can play an important role in enhancing umami flavor (33). As shown in Figure 2, five 5′-nucleotides were detected in three samples after 5.5 h of stewing. It can be seen that cooking mode significantly affected the levels of the 5′-nucleotides. 5′-AMP was the most abundant 5′-nucleotide in chicken soup (144.85–212.82 mg/L). Qi et al. (34) reported that among the 5′-nucleotides, the IMP is the most important fresh-tasting nucleotide in the chicken soup. In addition, the 5′-IMP in the CP and EC groups (67.02 and 68.08 mg/L) were higher than that in the SS group (60.89 mg/L, *P* < 0.05). 5′-GMP provided a meaty flavor and was a flavor enhancer, remarkably stronger than MSG (35). The CP group had the highest content of 5′-GMP at 53.83 mg/L, which was 1.67 times higher than that of the SS group (32.19 mg/L). And the content of five 5′-nucleotides in the CP group (325.53 mg/L) was significantly higher than that in the SS and EC group (291.23 and 265.03 mg/L, *P* < 0.05).

### The content of fatty acids

As the important precursors of meat flavor, FAs were associated with the characteristic flavor of meat soup (36). A total of 23 FAs were detected in chicken soups, including 10 saturated fatty acids (SFAs), 6 monounsaturated fatty acids (MUFAs), and 7 polyunsaturated fatty acids (PUFAs) (Table 1). The content of SFAs in SS soup (8429.82 μg/mL, 33.7%) was the highest compared with CP group (2706.84 μg/mL, 33.5%) and EC group (1502.67 μg/mL, 37.3%). SFAs, especially C12:0 and C14:0, had significant lipid-raising effects, promoting atherosclerosis by increasing blood lipid, total cholesterol and low-density lipoprotein (LDP) levels (37). The content of C12:0 and C14:0 in SS soup (15.23 and 225.74 μg/mL) were also significantly higher than the other two soups (*P* < 0.05). In our study, the UFAs account for 62.6 ∼ 66.5% of the total FAs and the main MUFAs in chicken soup was C18:1n9. Qian et al. (20) also reported that C18:1n9 was easy to migrate into to the Tuna (*Thunnus obesus*) head soup under higher temperature with the higher levels in the soup. Our study also found that the main PUFAs were C18:2n6, C18:3, C20:4n6, which was supported by the research of Zhang et al. (38), their study on the lipidomics of tuna soup showed that these three FAs were also the main FAs in soup and influenced effectively the metabolism of lipids. Compared with CP soup and EC soup, SS soup has the highest content of UFAs, which are prone to oxidation and form an unpleasant flavor that affects the taste of chicken soup. Overall, SS group had the highest fatty acid content at 24995.33 μg/mL, which significantly (*P* < 0.05) higher than the content in CP group (8097.79 μg/mL) and EC group (4036.54 μg/mL). It is since SS group has the highest content of lipid and can produce more FAs under a series of oxidative decomposition reactions at high temperatures.

**TABLE 1:** Effect of cooking mode on fatty acids of *Clitocybe squamulose* chicken soup.

	μg/mL	SS	CP	EC
SFAs	C4:0	18.19 ± 1.42^b^	30.97 ± 1.94^a^	32.76 ± 3.05^a^
	C6:0	6.23 ± 0.50^a^	3.06 ± 0.10^b^	1.71 ± 0.16^c^
	C10:0	6.49 ± 0.53^a^	1.33 ± 0.27^b^	0.89 ± 0.71^b^
	C12:0	15.23 ± 0.81^a^	3.88 ± 1.08^b^	1.85 ± 0.64^b^
	C14:0	225.74 ± 16.54^a^	83.94 ± 31.54^b^	39.61 ± 7.42^b^
	C15:0	18.46 ± 1.60^a^	9.45 ± 3.64^b^	3.03 ± 0.63c
	C16:0	6166.33 ± 510.37^a^	1989.12 ± 784.43^b^	1088.24 ± 213.63^b^
	C17:0	29.61 ± 1.50^a^	9.84 ± 4.28^b^	4.80 ± 0.74^b^
	C18:0	1868.16 ± 160.66^a^	539.80 ± 220.88^b^	317.91 ± 62.93^b^
	C20:0	75.37 ± 6.80^a^	35.45 ± 14.59^b^	11.86 ± 3.74^b^
	Total	8429.82 ± 697.61^a^	2706.84 ± 1058.86^b^	1502.67 ± 276.65^b^
MUFAs	C14:1	16.21 ± 1.13^a^	10.16 ± 3.69^b^	4.45 ± 1.38^b^
	C16:1	617.67 ± 45.93^a^	330.92 ± 127.78^b^	140.48 ± 22.37^b^
	C17:1	13.34 ± 1.00^a^	6.37 ± 2.93^b^	4.11 ± 1.38^b^
	C18:1n9	12085.92 ± 978.98^a^	4033.17 ± 1624.48^b^	1943.92 ± 458.37^b^
	C20:1	108.97 ± 5.52^a^	41.11 ± 16.40^b^	15.16 ± 3.39^c^
	C22:1	12.71 ± 0.77^a^	5.85 ± 1.70^b^	2.29 ± 2.08^b^
	Total	12854.82 ± 103.07^a^	4427.58 ± 1776.74^b^	2110.40 ± 478.40^b^
PUFAs	C18:2n6	3641.63 ± 272.27^a^	935.20 ± 363.15^b^	408.40 ± 84.78^b^
	C18:3	10.58 ± 1.16^a^	3.61 ± 1.23^b^	1.86 ± 0.42^b^
	C20:2	17.17 ± 0.95^a^	7.98 ± 2.78^b^	3.28 ± 0.46^c^
	C20:3	1.22 ± 0.85^a^	1.37 ± 0.43^a^	1.49 ± 0.37^a^
	C20:3n	0.55 ± 0.13^a^	0.56 ± 0.52^a^	1.31 ± 0.62^a^
	C20:4n6	36.40 ± 2.81^a^	12.92 ± 5.55^b^	5.04 ± 1.82^b^
	C22:6n3	3.14 ± 2.20^a^	1.74 ± 0.83^a^	2.09 ± 1.32^a^
	Total	3710.70 ± 278.19^a^	963.37 ± 373.22^b^	423.48 ± 86.23^b^
	Total FAs	24995.33 ± 2006.91^a^	8097.79 ± 3208.72^b^	4036.54 ± 838.35^b^

SS, *Clitocybe squamulose* chicken soup prepared in stainless-steel pot mode; CP, *Clitocybe squamulose* chicken soup prepared in ceramic pot mode; EC, *Clitocybe squamulose* chicken soup prepared in electrical ceramic stewpot mode. The lowercase letters show differences between cooking modes per time point.

### Sensory evaluation

**FIGURE 3:**
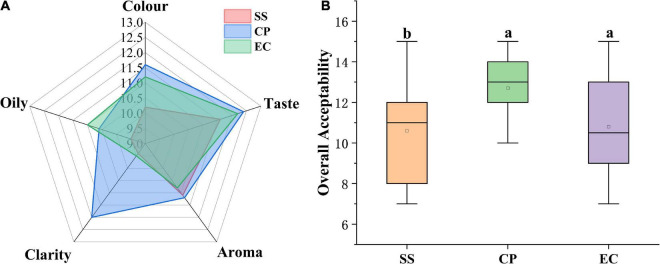
Radar chart of sensory evaluation **(A)** and boxplot of overall acceptability **(B)** of *Clitocybe squamulose* chicken soup. The different lowercase letters denote statistical significance.

**FIGURE 4:**
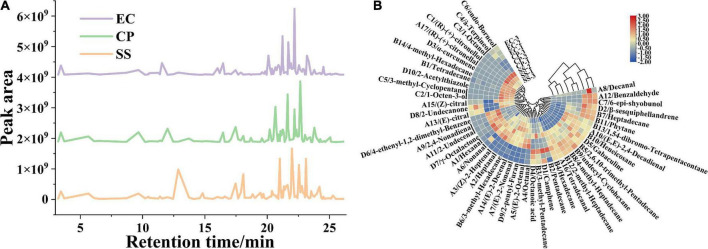
Effects of cooking mode on the area of peaks for flavor substances from the *Clitocybe squamulose* chicken soup **(A)**, and heat map obtained from cluster analysis of flavor substances in *Clitocybe squamulose* chicken soup with different cooking mode **(B)**. The color in heat map represented the relative concentration of flavor substance, the gradual color change from red to blue indicated the concentration change from high to low.

The sensory evaluation results are presented in Figure 3, among the three chicken soups, revealing that cooking modes significantly affected the chicken soup sensory. CP group showed the highest values in color (11.60), taste (12.40), aroma (11.20), clarity (12.00), and over acceptability (12.40) (Figure 4A). In terms of the sensory color of the chicken soup, the CP group presented a pleasant golden color, while the EC samples exhibited a dark brown color, and the SS samples displayed a whitish color, both of which had lower color scores than CP sample. The taste in EC group scored 12.20, which was close to that in CP (12.40) group with strong mellow taste and rich umami. The SS soup taste scored (11.60) lower than the other two soups, but not significantly different from them (*P* > 0.05). The CP and SS soups exhibited higher aroma scores (11.20 and 11.10, respectively) than EC soup, but no significant difference in aroma was observed among three soups (*P* > 0.05), all of which presented specific *Clitocybe squamulose* aroma and the fresh meat-like aroma. In terms of clarity, EC and SS soups were significantly more turbid than CP soup (*P* < 0.05). In addition, oily is another important sensory quality index for chicken soup. After the chicken meat has been stewing for a long time, the lipid is gradually dissolved into the soup to form a grease layer on the surface of the chicken soup, which emits the unique aroma of chicken soup. However, excessive oil and fat will cause an unpleasant taste of excessive greasiness. SS soup showed the lowest scores in oily, which might be due to that SS was heated unevenly, resulting in the large fat particle distribution and poor ability to dissolve into the soup. Furthermore, the assessment of the overall acceptability showed that the CP sample exhibited the highest total score, followed by EC and SS samples (Figure 4B, *P* < 0.05).

### The content of volatile compounds

The production of volatile flavor compounds is related to multiple factors such as the nutrients of the raw materials, the cookware, the cooking time and cooking mode (6, 7). Our data indicated that the flavor substances were significantly different among the 3 groups (*P* < 0.05). The types and relative concentrations of volatile compounds were shown in Figure 4A. A total of 48 volatile compounds were isolated and identified by SPME-GC-MS in chicken soup. The differences in the concentrations of volatile flavor compounds among the three groups were significant with the total concentration of volatile flavor compounds in CP and EC samples (1397.70 μg/kg, 1591.73 μg/kg) significantly higher (*P* < 0.05) than that in SS sample (742.07 μg/kg) (Figure 4B and Supplementary Table 1).

Aldehyde is produced through the oxidation and degradation of lipids (8), and the concentration of aldehyde is increased with lipid dissolution during the cooking process. A total of 17 aldehydes were identified from the 3 chicken soups cooked in different cooking modes with each soup varied in aldehyde type and concentration from one soup to another. The concentration of 15 aldehydes reached up to 295.50 μg/kg in CP group, which was significantly higher in concentration and more in types than that in EC group (12 aldehydes, 245.96 μg/kg), and followed by SS group (12 aldehydes, 243.30 μg/kg). The concentrations of aroma-active compounds of hexanal (A1), heptanal (A2), (Z)-2-heptenal (A3), octanal (A4), (E)-2-octenal (A5), nonanal (A6), 2,4-nonadienal (A9), (E, E)-2,4-decadienal (A10), 2-undecenal (A11), and tetra-decanal (A16) were higher in CP samples than in SS and EC samples. In addition, these aldehyde compounds have been considered as a cause of the chicken aroma, since the removal of these compounds from the volatile substances results in a loss of the chicken aroma (39). A1 and A6 was reported as the key flavor substance, contributing to the intense grass-like aroma note, and oily aroma of chicken soup (40, 41). This showed that the CP soup had an advantage in contributing to more pleasant fatty and grass aroma to the overall flavor. A1 and A6 were also reported to be characteristic flavor substances in the Chinese smoked chicken (42). Benzaldehyde (A12) is the aromatic aldehyde detected in chicken soup samples with almond and nut flavor, and benzaldehyde is generated from Strecker degradation of phenylalanine or a linolenic acid oxidation (43, 44). In this study, terpene aldehydes such as (E)-citral (A13) and (Z)-citral (A15) were also founded in chicken soup. We speculated that the above volatile aldehydes might be potential contributors to the *Clitocybe squamulose* chicken soup flavor, owing to their low flavor thresholds and unique flavor (45).

The alkanes are generated from the lysis and degradation of fatty acid. Most alkane compounds possess a long carbon chain, therefore they contribute little to the aroma and taste of the soup, but they are essential flavor coordinators. In this study, the chicken soup samples were added with *Clitocybe squamulose*, and related studies showed that the volatile compounds of edible fungi contain large amount of high-threshold alkanes (46). Our data indicated that alkanes were the most abundant volatile components in all three samples (414.68 μg/kg for SS samples, 984.77 μg/kg for CP samples, and 1197.63 μg/kg for EC samples). Multiple types of alkanes presented a concentration above 100 μg/kg, such as pentadecane (B2), hexadecane (B4), 2,6,10-trimethyl-pentadecane (B5), and heptadecane (B7), and resulted in a positive effect on the overall flavor of the chicken soup.

A total of 7 types of alcohols were identified from the chicken soup. Five types identified alcohols were presented in the SS, accounting for 71% of the identified alcohols, while the other two samples contained only 29% (CP, 2 type) and 43% (EC, 3 types) of the identified alcohols, respectively. Among these, low concentration of 1-octen-3-ol was detected only in CP sample. 1-octen-3-ol (C2, unsaturated alcohol derived from linoleic acid oxidation) is regarded as a key flavor compound due to its low threshold (0.001 mg/kg) (47). C2 is a higher alcohol, also known as mushroom alcohol, and it is associated with mushroom, earthy, grassy, oily, vegetative aroma (48), major composition in cosmetic and food flavors (49). Furan contributes significantly to the soup aroma (44). The 2-penty-1-furan (D9), as furan compound, was detected from CP and SS samples with the concentration of 3.24 and 3.12 μg/kg, respectively, but it was not detected from EC sample.

### Multivariate statistical analysis

#### Differences in volatile compound composition based on principal component analysis and orthogonal partial least squares discrimination analysis

**FIGURE 5:**
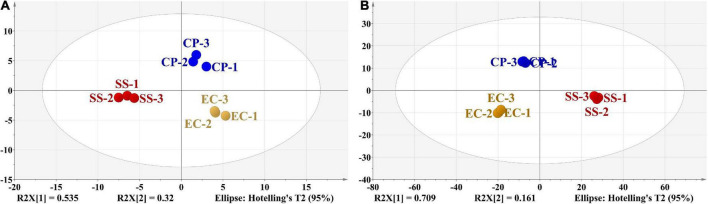
PCA of detected volatile flavor compounds **(A)**. OPLS-DA of detected volatile flavor compounds **(B)**. Circles of different colors represented chicken soups with different cooking modes, and the distance between the points reflected the similarity of their volatile components.

Multivariate statistical analysis and relevant models were confirmed to be effective in evaluating and verifying food composition (19). The high values of *R*^2^ (X) (0.855) and Q^2^ (0.734) obtained from PCA analysis of 48 volatile components confirmed the validity of the PCA model. Two extracted PCs (eigenvalues > 1) explained 85.5% of the total variance with PC1 and PC2 accounting for 53.5% and 32.0% of the total variance, respectively. The score plot (Figure 5A) showed that all the samples were well clustered into three characteristic groups, indicating the good distribution of samples in the space (50, 51). For PC1 vector, significant differences existed between these three groups. SS samples were located on the negative side of PC1 axis while EC and CP samples were located on the positive side of PC1. CP samples were located on the positive side of PC2 axis; EC and SS samples was located on the negative side of PC2 axis. Therefore, PCA showed an evident separation trend among CP soup, EC soup and SS soup. However, the noise interference could not be eliminated from PCA. To achieve more accurate classification and identification results, the supervised discriminant method OPLS-DA was further applied for sample analysis (52).

OPLS-DA, as a multivariate calibration method, can be used to reduce the dimensionality of the dataset (37, 53). In this study, OPLS-DA was applied to reveal the correlation between cooking mode with different cooking mode and the concentration of volatile compounds in chicken soup. In OPLS-DA model, the fit parameters *R*^2^ (X), *R*^2^ (Y), and *Q*^2^ were found to be 0.964, 0.998, and 0.980, respectively, indicating a good fitness and acceptable predictability of the OPLS-DA model. Figure 5B showed that the three types of chicken soups were more clearly distinguished in the OPLS-DA model than the results from the PCA. CP and EC groups were located on the negative side of PC1 axis, but SS group were located on the positive side. And CP samples were located on the positive side of PC2 axis alone. In addition, within each group, chicken soup samples exhibited an aggregation trend, and between groups, three different types of samples displayed a separation trend from each other.

#### Analysis of the differences in important volatile components of *Clitocybe squamulose* chicken soup with different cooking methods

**FIGURE 6:**
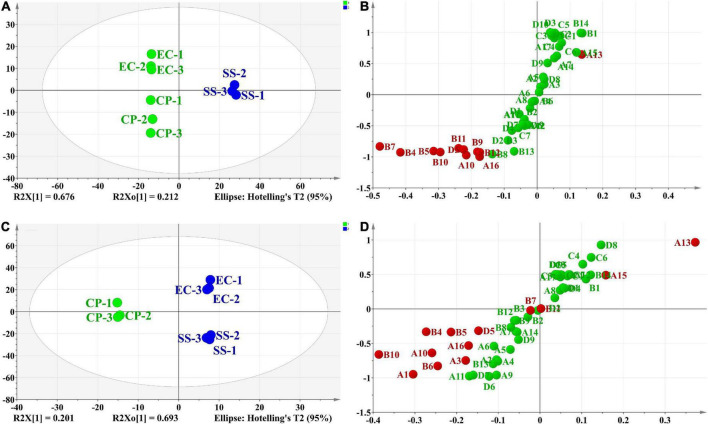
Score scatter plot **(A,C)** and S-plot **(B,D)** of differential flavor compounds in comparison of SS vs. CP and EC, CP vs. EC and SS based on OPLS-DA.

**FIGURE 7:**
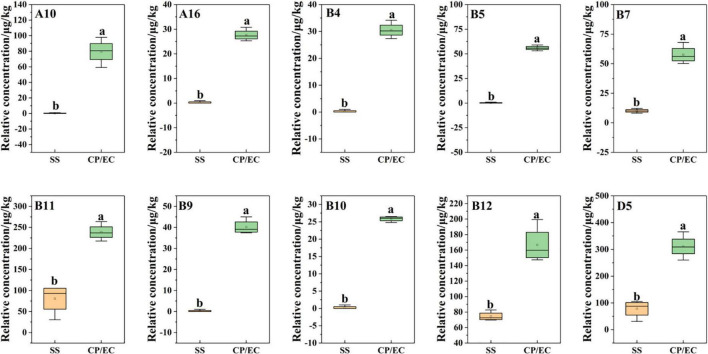
Statistical analysis of differential flavor compounds related to *Clitocybe squamulose* chicken soup of SS vs. CP/EC group.

**FIGURE 8:**
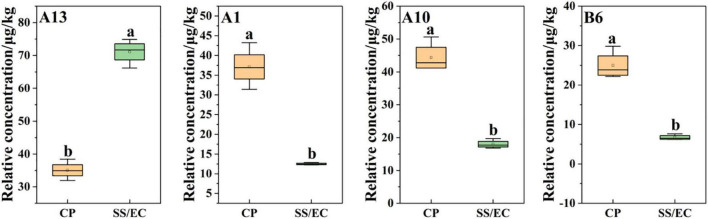
Statistical analysis of differential flavor compounds related to *Clitocybe squamulose* chicken soup of CP vs. SS/EC group.

**FIGURE 9:**
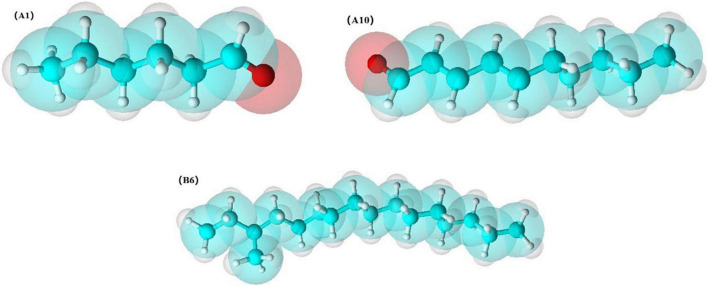
Molecular structure diagram of hexanal, (E,E)-2,4-decadienal and 3-methyl-hexadecane.

In addition, based on the OPLS-DA results, was applied to distinguish the SS and CP groups from the other samples, respectively, and to screen out the differential substances (Figures 6A,C). As shown in S-plot, 48 volatile compounds were scattered (Figures 6B,D), and the variables with VIP > 1 and p (corr) > 0.8 have significant differences between categories and play an important role in classification (54). A total of 10 flavor substances were screened for differences between SS chicken soup and the other 2 types of chicken soup based on the above principles, as shown in Figure 7. (E,E)-2,4-decadienal (A10), (A16), hexadecane (B4), 2,6,10-trimethyl-pentadecane (B5), heptadecane (B7), (B9), heneicosane (B10), phytane (B11), (B12) and gabaculine (D5) were the 10 differential volatile components. Both traditional CP s and EC s are composed of clay and have a more similar composition, while SS s are composed of dense metal. The difference in the material of the cookware results in SS chicken soup having more differentiated flavor substances compared to the other two types of chicken soup. Compared to the other two chicken soups, there were 4 differential flavor substances in CP chicken soup (Figure 8), namely hexanal (A1), A10, (E)-citral (A13), and 3-methyl-hexadecane (B6). Among them, A13 was significantly lower in CP than in EC and SS groups, while A1, A10 and B6 were significantly higher in CP than in the other groups, so these three substances were identified as the characteristic flavor compounds for CP groups. The molecular structures of A1, A10 and B6 are illustrated in Figure 9. B6 is a saturated alkane, mainly produced by the pyrolysis of lipids (46), which coordinates the overall flavor of the chicken soup. A1 contains an aldehyde group and the substance has a raw fatty flavor, accompanied by characteristic aromas such as grassy and apple, and is often used as a food flavor (55). Zeng et al. (8) has reported that A1 has been detected as a key flavor substance in chicken soup cooked in CP. It is evident that A1 was not only an important flavor substance in chicken soup, but also a characteristic differential substance that distinguishes CP cooking of soup from other cooking modes. A10 contains an aldehyde group and two unsaturated olefin bonds. The substance has a strong chicken flavor and chicken lipid flavor (56) and plays a key role in the formation of the flavor of chicken soup. A10 was also reported as a flavor active substance of chicken soup in Zhang et al.’s study (15) which was similar to our findings. The levels of A10 in CP group were significantly higher than EC group (*P* < 0.05).

## Conclusion

In this study, the *Clitocybe Squamulose* chicken soup was cooked with SS, CP and EC, respectively, and the results showed that CP soup exhibited a higher content of soluble solids, total sugar, total protein and 5′-nucleotides than the other two soups (SS and EC) and obtained the highest scores in the sensory evaluation. 48 volatile flavor compounds, mainly aldehydes and alkanes, were identified by SPME-GC-MS. The OPLS-DA revealed that hexanal, (E,E)-2,4-decadienal, and 3-methyl- contributed to the characteristic flavor profile of CP group. These results indicated the effect of cooking mode on the quality of the chicken soup. It could be concluded that using the CP mode might be an optimal mode for chicken soup cooking. The cookware in the EC and CP groups was made of the same ceramic, but there was a difference in the boiling time of the chicken soup, resulting in a poorer quality EC sample. Hence improving the design of the heating program in EC may improve the quality of chicken soup, which also informs cookware manufacturers.

## Data availability statement

The original contributions presented in this study are included in the article/Supplementary material, further inquiries can be directed to the corresponding author.

## Author contributions

JL: methodology, investigation, data curation, and writing—original draft. RW and JW: methodology and writing—review and editing. YW and XZ: software and investigation. LZ: visualization and resources. YZ: conceptualization, funding acquisition, supervision, and writing—review and editing. All authors read and agreed to the published version of the manuscript.
