# Supplementary material for: Effect of cooking modes on quality and flavor characteristic in Clitocybe squamulose chicken soup
Source: Front Nutr. 2022 Nov 15;9:1048352. doi: 10.3389/fnut.2022.1048352 (PMC9705982; doi:10.3389/fnut.2022.1048352)
Supplement: Supplementary file 1 [file Data_Sheet_1.pdf]

Supplementary Material

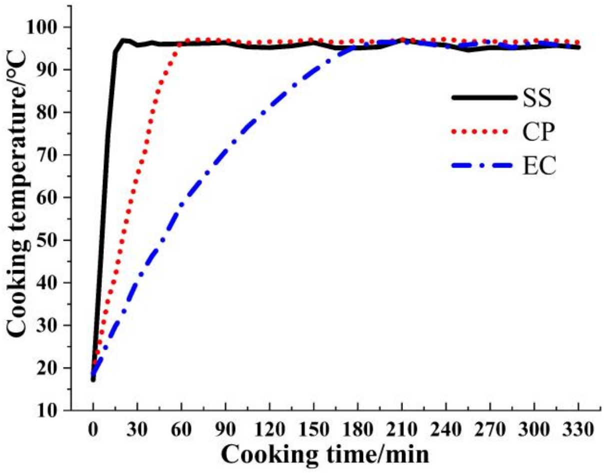

Figure S1. Effect of cooking mode on cooking time and temperature of *Clitocybe squamulose* chicken soup. SS, *Clitocybe squamulose* chicken soup prepared in stainless-steel pot mode; CP, *Clitocybe squamulose* chicken soup prepared in ceramic pot mode; EC, *Clitocybe squamulose* chicken soup prepared in electrical ceramic stewpot mode.

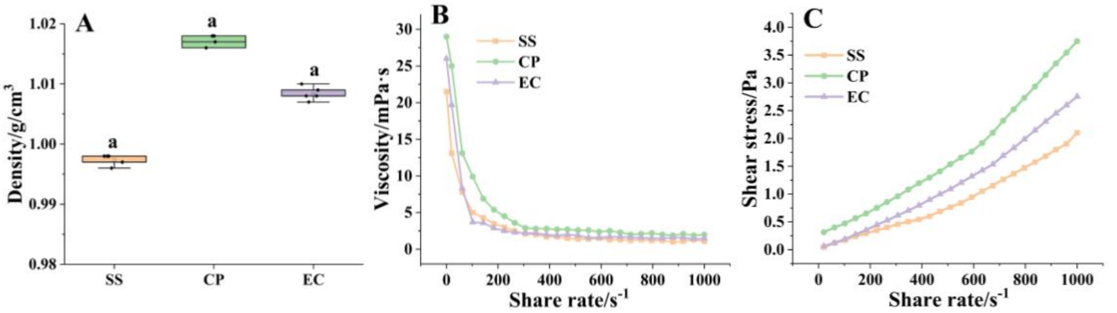

Figure S2. Effect of cooking mode on density **(A)**, viscosity **(B)**, and shear stress **(C)** of *Clitocybe squamulose* chicken soup.

Table S1. Identification and quantification of volatile flavour compounds in 3 types of *Clitocybe squamulose* chicken soups.

NO.	Compound name	CAS	Identification	Relative concentration (μg/kg)		
				SS	CP	EC
A1	Hexanal	66-25-1	MS, RI	15.34±1.35^{b}	37.13±5.93^{a}	9.65±1.48^{b}
A2	Heptanal	111-71-7	MS, RI	3.33±0.36^{a}	5.38±1.71^{a}	ND
A3	(Z)-2-Heptenal	57266-86-1	MS, RI	10.27±1.99^{b}	15.81±2.56^{a}	ND
A4	Octanal	124-13-0	MS, RI	6.86±1.22^{ab}	9.48±2.18^{a}	5.19±0.17^{b}
A5	(E)-2-Octenal	2548-87-0	MS, RI	5.63±0.92^{a}	6.40±1.29^{a}	2.79±0.17^{a}
A6	Nonanal	124-19-6	MS, RI	21.92±6.08^{a}	26.56±1.46^{a}	19.63±5.76^{b}
A7	(E)-2-Nonenal	18829-56-6	MS, RI	8.16±1.87^{a}	6.68±0.30^{a}	ND
A8	Decanal	112-31-2	MS, RI	3.00±0.99^{a}	2.75±0.52^{a}	6.78±6.95a
A9	2,4-Nonadienal	6750-03-4	MS, RI	ND	2.83±0.87^{a}	ND
A10	(E,E)-2,4-Decadienal	25152-84-5	MS, RI	ND	44.35±5.43^{a}	35.98±3.06^{a}
A11	2-Undecenal	2463-77-6	MS, RI	16.38±1.24^{b}	23.63±0.58^{a}	16.03±0.74^{b}
A12	Benzaldehyde	100-52-7	MS, RI	ND	ND	5.10±0.61^{a}
A13	(E)-citral	141-27-5	MS, RI	75.17±6.38^{a}	35.08±3.28^{b}	67.04±3.18^{a}
A14	(E)-2-Decenal	3913-81-3	MS, RI	19.43±2.03^{a}	18.36±0.60^{a}	12.67±0.89^{b}
A15	(Z)-citral	106-26-3	MS, RI	55.27±3.68^{a}	34.57±18.39^{a}	39.87±1.32^{a}
A16	Tetradecanal	124-25-4	MS, RI	ND	26.47±1.85^{a}	25.24±0.87^{a}
A17	(R)-(+)-citronellal	2385-77-5	MS, RI	2.54±1.21^{a}	ND	ND
				243.30±24.51^{b}	295.50±16.20^{a}	245.96±14.05^{b}
B1	Tetradecane	629-59-4	MS, RI	23.72±0.72^{a}	8.21±0.95^{b}	6.82±1.87^{b}
B2	Pentadecane	629-62-9	MS, RI	102.28±4.25^{a}	103.94±6.43^{a}	104.41±13.61^{a}
B3	3-methyl-Pentadecane	2882-96-4	MS, RI	27.45±2.53^{a}	34.47±10.35^{a}	37.88±5.56^{a}
B4	Hexadecane	544-76-3	MS, RI	80.36±43.22^{b}	224.12±25.93^{a}	253.34±24.67^{a}
B5	2,6,10-trimethyl-Pentadecane	3892-00-0	MS, RI	74.42±7.24^{b}	158.43±33.18^{a}	174.70±23.96^{a}
B6	3-methyl-Hexadecane	6418-43-5	MS, RI	13.40±1.62^{b}	24.94±4.24^{a}	ND
B7	Heptadecane	629-78-7	MS, RI	77.91±40.98^{c}	237.19±75.97^{b}	384.52±30.53^{a}
B8	4-methyl-Heptadecane	26429-11-8	MS, RI	ND	14.07±2.42^{b}	18.91±1.08^{a}
B9	undecyl-Cyclohexane	54105-66-7	MS, RI	ND	23.94±3.59^{b}	37.04±5.83^{a}
B10	Heneicosane	629-94-7	MS, RI	ND	90.91±21.40^{a}	68.24±17.69^{b}
B11	Phytane	638-36-8	MS, RI	ND	37.11±2.45^{b}	74.77±4.09^{a}
B12	2-methyl-Heptadecane	1560-89-0	MS, RI	ND	21.72±2.37^{b}	33.56±3.70^{a}
B13	1,54-dibromo-Tetrapentacontane	-	MS, RI	ND	5.72±0.46^{a}	3.43±0.49^{b}
B14	4-methyl-Hexadecane	25117-26-4	MS, RI	15.14±1.67^{a}	ND	ND
				414.68±44.67^{b}	984.77±165.33^{a}	1197.63±112.42^{a}
C1	(R)-(+)-citronellol	1117-61-9	MS, RI	4.43±1.57^{a}	ND	ND
C2	1-Octen-3-ol	1394	MS, RI	ND	2.73±0.65^{a}	ND
C3	1-Octanol	111-87-5	MS, RI	1.72±0.58^{a}	ND	ND
C4	à-Terpineol	98-55-5	MS, RI	10.38±3.19^{a}	4.46±1.05^{b}	6.61±1.33^{ab}

C5	3-methyl-Cyclopentanol	18729-48-1	MS, RI	2.50±0.34[a]	ND	ND
C6	endo-Borneol	507-70-0	MS, RI	7.08±2.78[a]	ND	2.92±0.48[b]
C7	6-epi-shyobunol	-	MS, RI	ND	ND	4.84±0.36[a]
				26.11±8.41[a]	7.18±1.13[b]	14.37±1.68[b]
D1	Camphene	79-92-5	MS, RI	3.53±0.59[a]	4.03±1.58[a]	9.49±5.53[a]
D2	β-sesquiphellandrene	20307-83-9	MS, RI	21.07±0.74[b]	25.81±1.23[b]	34.47±3.55[a]
D3	α-curcumene	644-30-4	MS, RI	12.10±1.62a	13.65±3.95[b]	20.91±2.97[b]
D4	Octanoic acid	124-07-2	MS, RI	ND	6.71±2.01[a]	ND
D5	Gabaculine	87980-11-8	MS, RI	10.06±2.02[b]	53.11±22.64[a]	62.19±9.03[a]
D6	4-ethenyl-1,2-dimethyl-Benzene	27831-13-6	MS, RI	1.36±0.03[a]	ND	ND
D7	γ-Octalactone	104-50-7	MS, RI	ND	3.81±0.82[a]	ND
D8	2-Undecanone	112-12-9	MS, RI	4.92±0.93[a]	ND	6.69±1.59[a]
D9	2-pentyl-Furan	3777-69-3	MS, RI	3.24±0.87[a]	3.12±0.30[a]	ND
D10	2-Acetylthiazole	24295-03-2	MS, RI	1.68±0.13[a]	ND	ND
				57.97±3.76[b]	110.25±23.86[a]	133.76±9.91[a]
				742.07±77.04[b]	1397.70±181.25[a]	1591.72±116.87[a]

a-c, Lower-case letters within the same row indicate significant differences ($P < 0.05$); ND, not detected; "-", not found. A, aldehydes volatile compounds; B, alkanes volatile compounds; C, alcohols volatile compounds; D, others volatile compounds.

Table S2. The differences between this work and previous work in fatty acids on chicken soup.

Fatty acids/mg/mL		This work			Previous work (Li ZY, 2022)		
		SS	CP	EC	GCS	HCS	CBS
SFAs	C4:0	0.018±0.001^{b}	0.031±0.002^{a}	0.033±0.003^{a}	ND	ND	ND
	C8:0	ND	ND	ND	0.03±0.001	0.02±0.002	0.02±0.002
	C6:0	0.006±0.001^{a}	0.003±0.000^{b}	0.002±0.000^{c}	ND	ND	ND
	C10:0	0.006±0.001^{a}	0.001±0.000^{b}	0.001±0.001^{b}	0.13±0.002	0.07±0.002	0.07±0.002
	C12:0	0.015±0.001^{a}	0.004±0.001^{b}	0.002±0.001^{b}	ND	ND	ND
	C14:0	0.226±0.017^{a}	0.084±0.032^{b}	0.040±0.007^{b}	0.13±0.004	0.06±0.001	0.07±0.001
	C15:0	0.018±0.002^{a}	0.009±0.004^{b}	0.003±0.001c	ND	ND	ND
	C16:0	6.166±0.510^{a}	1.989±0.784^{b}	1.088±0.214^{b}	4.12±0.039	1.97±0.03	3.22±0.015
	C17:0	0.030±0.002^{a}	0.010±0.004^{b}	0.005±0.001^{b}	ND	ND	ND
	C18:0	1.868±0.161^{a}	0.540±0.221^{b}	0.318±0.063^{b}	0.99±0.020	0.50±0.003	1.05±0.009
	C20:0	0.075±0.007^{a}	0.035±0.015^{b}	0.012±0.004^{b}	ND	ND	ND
	Total	8.430±0.698^{a}	2.707±1.059^{b}	1.503±0.277^{b}	5.40±0.066	2.72±0.039	4.43±0.029
MUFAs	C14:1	0.016±0.001^{a}	0.010±0.004^{b}	0.005±0.001^{b}	ND	ND	ND
	C16:1	0.618±0.046^{a}	0.331±0.128^{b}	0.140±0.022^{b}	1.12±0.012	0.51±0.04	0.42±0.026
	C17:1	0.013±0.001^{a}	0.006±0.003^{b}	0.004±0.001^{b}	ND	ND	ND
	C18:1n9	12.086±0.979^{a}	4.033±1.624^{b}	1.944±0.458^{b}	7.64±0.071	3.85±0.038	4.59±0.014
	C20:1	0.109±0.006^{a}	0.041±0.016^{b}	0.015±0.003^{c}	ND	ND	ND
	C22:1	0.013±0.001^{a}	0.006±0.002^{b}	0.002±0.002^{b}	ND	ND	ND
	Total	12.855±0.103^{a}	4.428±1.777^{b}	2.110±0.478^{b}	8.76±0.083	4.36±0.078	5.01±0.040
PUFA	C18:2n6	3.642±0.272^{a}	0.935±0.363^{b}	0.408±0.085^{b}	2.53±0.029	2.25±0.010	4.07±0.021
	C18:3	0.011±0.001^{a}	0.004±0.001^{b}	0.002±0.000^{b}	0.14±0.005	ND	0.29±0.005
	C20:2	0.018±0.001^{a}	0.008±0.003^{b}	0.003±0.000^{c}	ND	ND	ND
	C20:3	0.001±0.001^{a}	0.001±0.001^{a}	0.001±0.000^{a}	ND	ND	ND
	C20:3n6	0.001±0.000^{a}	0.001±0.001^{a}	0.001±0.001^{a}	ND	ND	ND
	C20:4n6	0.036±2.81^{a}	0.013±0.006^{b}	0.005±0.002^{b}	ND	ND	ND
	C22:6n3	0.003±2.20^{a}	0.002±0.001^{a}	0.002±0.001^{a}	ND	ND	ND
	Total	3.711±278.19^{a}	0.963±0.373^{b}	0.423±0.086^{b}	2.6730.034	2.25±0.010	4.36±0.026
	TFAs	24.995±2.007^{a}	8.098±3.209^{b}	4.037±0.838^{b}	16.83±0.183	9.33±0.127	13.80±0.095

ND, not detected; CBS, Cobb broiler soup; GCS, Gushi chicken soup; HCS, Honglashan chicken soup.

Li ZY, Li XM, Cai ZX, Jin GF, Immunomodulatory effects of chicken soups prepared with the native cage-free chickens and the commercial caged broilers. *Poultry Science,* (2022) 101(10): 102053.

Table S3. Fitting parameters for temperature variation with time for each cooking mode.

Cooking mode	Fitting parameters (y: cooking temperature/°C, x: cooking time/min)		R^2
SS	y=5.204x+18.770	(0 min < x < 15min)	0.99318
	y=95.74	(15 min < x < 330 min)	
CP	y=1.361x+22.085	(0 min < x < 60 min)	0.98515
	y=96.75	(60 min < x < 330 min)	
EC	$y=9.127 \cdot x^{0.451}$	(0 min < x < 180 min)	0.98783
	y=96.00	(180 min < x < 330 min)	

Table S4. The differences between *Clitocybe squamulose* chicken soup and chicken soup on soluble solid matter, total sugar, crude protein and overall acceptability (sensory evaluation).

	Soluble solid matter/ g/100mL	Total sugar/ mg/mL	Crude protein/ g/100g	overall acceptability/ 15-point scale
Chicken soup	2.89	1.10	5.66	9.59
SS	3.92	1.65	6.76	10.6
CP	5.83	2.38	7.58	12.4
EC	4.43	2.26	7.51	10.8

Determination of density

The density of the soup samples was measured using a handheld digital densitometer (DMA 35, Anton Paar, Denmark) at 25°C. The result is expressed as g/cm^3.

Rheological assay

The viscosity and viscoelasticity of the samples were measured using a Malvern Rotating Rheometer (MAL1038384, Kinexus Prot, Malvern Instrument Co., Ltd., Malvern, UK). The temperature was fixed at 25°C during the measurements with an accuracy of ±0.1°C. The samples were placed on the measuring plate and left for 5 min because of structure recovery and temperature equilibrium. The emulsion samples were placed at the 60 mm diameter parallel plate geometry, and the geometry gap was set at 0.5 mm. The viscosity was measured by a steady shear mode with a shear rate from 0.1 to 1000 s^{-1}. A strain sweep was performed to determine the viscoelastic behavior of the samples. The results are expressed in mPaS^{-1}. A thin layer of silicone oil was applied to the exposed edges of the sample to prevent water evaporation.

Density

As shown in Figure S2A, the density of chicken soups ranged from 0.818 to 1.008. Although the densities of the CP and EC groups were higher than that in the SS group, there was not significantly difference affected by the cooking modes ($P > 0.05$).

Rheological characteristics

The viscosity is an important physical and chemical index for evaluating the emulsion. The viscosity values in the soups are illustrated in Figure S2B. According to Stokes' law, the greater viscosity of the emulsion enhances the stability of the emulsion. The viscosity of all groups decreased when the shear rate increased from 0.1 to 100 s^{-1}; then levelled off at shear rates of 100 to 1000 s^{-1}. This result indicated that the soup exhibited shear thinning behavior of non Newtonian fluid properties (1). This characteristic may be due to the application of shear stress, which cause the oil droplets in the flocculated state to separate from each other during the shearing process, resulting in rearrangement of the micro structure in the fluid (2-4). Among all the samples, viscosity curves of the SS and EC samples were similar and they became closer with the shear rate increasing. The viscosity of the CP group was higher compared to the SS and EC groups ($P < 0.05$), suggesting that CP mode has the effective emulsion ability, the lipids and proteins in the CP soup were fully emulsified, thus the stability and viscosity were improved.

As shown in Figure S2C, the yield stress in the chicken soup was existed because the curve does not pass through the origin, suggesting that the interaction of macromolecules and the aggregation of particles in the chicken soup forming a dense network structure (5,6). The shear stress in three chicken soups rose with the shear rate increased, the CP soup manifested the higher shear stress value, followed by EC soup and SS soup. Therefore, the differences in cooking modes affected the protein content, lipid content and viscosity of the chicken soup system, then further lead to difference in shear stresses of the chicken soup.

Reference

(1) Lekjing S, Venkatachalam K, Wangbenmad C. Biochemical evaluation of novel seabass (Lates calcarifer) fish essence soup prepared by prolonged boiling process. *Arabian J Chem.* (2021) 14(10): 103365. doi: 10.1016/j.arabjc.2021.103365

(2) Zhang XW, Cheng Z, Zhao XY, Liu HK, Hu HF, Wang M, Guo JG, Effects of the oat β-glucan on the functional and structural properties of defatted walnut meal flour. *Food Chem Advances.* (2022) 100071. doi: 10.1016/j.focha.2022.100071

(3) Li D, Zhao Y, Wang X, Effects of (+)-catechin on a rice bran protein oil-in-water emulsion: Droplet size, zeta-potential, emulsifying properties, and rheological behavior. *Food Hydrocolloids*. (2020), 98(JAN). doi: 10.1016/j.foodhyd.2019.105306

(4) Li Y, Li J, Fan L. Effects of combined drying methods on physicochemical and rheological properties of instant Tremella fuciformis soup. *Food Chem.* (2022) 396. doi: 10.1016/j.foodchem.2022.133644

(5) Mondal IH, Rangan L, Uppaluri V S. A robust and novel methodology for the optimal targeting of leafy vegetable mix soup formulations. *Lwt.* (2020) 134: 110152. doi: 10.1016/j.lwt.2020.110152

(6) Wen D, Li L, Yan H, Effect of Ginger on Chemical Composition, Physical and Sensory Characteristics of Chicken Soup. *Foods.* (2021) 10(7). doi:10.3390/foods10071456
